# Managing TB infection among household contacts of patients with pulmonary TB

**DOI:** 10.5588/ijtldopen.26.0237

**Published:** 2026-10-12

**Authors:** A. Kalra, M. Das, A.S. ThekkePurakkal, T. Showket, S. Raj, S. Prakash, R. Dasari, H. Solanki, R. Rao, V. Dhawan, M.A. Yassin, S.S. Chadha, U.B. Singh

**Affiliations:** 1 FIND, New Delhi, India;; 2 Karnataka Health Promotion Trust, Bengaluru, Karnataka, India;; 3 TB Alert India, Secunderabad, Telangana, India;; 4 Central TB Division, WHO NTEP Technical Support Network, New Delhi, India;; 5 Central TB Division, Ministry of Health and Family Welfare, New Delhi, India;; 6 The Global Fund to Fight AIDS, Tuberculosis and Malaria, Geneva, Switzerland;; 7 FIND, Geneva, Switzerland.

**Keywords:** tuberculosis, India, interferon-gamma release assays, TPT, test and treat, symptom screening

## Abstract

**BACKGROUND:**

One third of India’s population has TB infection (TBI) with 10% at risk of developing TB disease. A multi-centric TBI initiative for household contacts (HHCs) of people with pulmonary TB (PTB) was implemented.

**METHODS:**

The project Joint Effort for Elimination of TB was implemented in collaboration with India’s National TB Elimination Programme from April 2021 to March 2024. This included doorstep ruling out of TB disease, screening HHCs for TBI, provision of TB preventive treatment (TPT), and adverse events management.

**RESULTS:**

Of the 151,890 notified patients with PTB, 138,812 (91%) could be contacted. Of these, 112,687 (74%) consented to home visits and 94,621 (63%) households were visited. During the visits, 403,923 HHCs were identified, of whom 398,822 (99%) were screened for TBI; 310,605 (77%) consented and 253,885 (63%) initiated on TPT. Adverse events were reported by 3% HHCs with no serious episodes. Of the eligible 148,715 HHCs on TPT, 130,304 (88%) completed with an increasing trend in the TPT completion.

**CONCLUSION:**

This community-based model for TBI management is feasible and effective. High screening and treatment completion rates alongside minimal adverse events prove this strategy is scalable and replicable for other high-burden settings worldwide.

Management of TB infection (TBI) has been recognised as an important measure towards TB elimination globally. An estimated 25% of the global population have TBI,^1^ and about 5%–10% of individuals with TBI may develop active TB disease during their lifetime^2^; however, the risk is much higher during the first 5 years.^3^ Among children, about 83% may develop TB disease within 3 months of exposure.^4^ A focus on TBI management aids in minimising TB incidence due to TB re-activation and prevent the individuals from developing TB disease.^5^ The WHO recommends TB preventive treatment (TPT) as a key intervention to achieve End TB Strategy targets and prioritises TPT for individuals with higher risk of developing TB including people living with HIV (PLHIV) and household contacts (HHCs) of patients diagnosed with bacteriologically confirmed pulmonary TB (PTB).^2,6^

Systematic reviews have reported outcomes of TBI management,^7^ and feasibility of TPT regimens for TBI among general population and those at higher risk of developing TB such as PLHIV and those with TB contact history.^8,9^ Adherence levels and completion of TPT have been found promising in previous studies^8,10^; however, information on addressing TBI country-wide and TPT experiences from low-and middle-income countries in Asia remains limited.^11^ India contributes to more than one fourth of the global TB cases. The country also has highest burden of TBI, accounting for 31% of crude TBI prevalence in adults aged more than 15 years.^12^ A systematic review estimated TBI pooled prevalence for India to be 41% with geographical and age-group variations.^13^ The National Strategic Plan for TB (2017–2025) recommended prioritisation of TPT provision to high-risk population including HHCs.^14^ The India’s National TB Elimination Programme (NTEP) launched TPT guidelines in 2021, that included strategies for addressing TBI and TPT provision for at-risk population.^15^ Studies in India have documented the need for scaling up TBI detection and TPT provision to individuals at risk of developing TB including HHCs.^16–18^ Though studies have reported appreciable TPT completion rates, these findings represent a limited geographical area.^17,19^ Since the provision of TPT to vulnerable population including HHCs is a newer initiative in India, nation-wide studies reflecting the TPT experiences in a programmatic setting are unavailable.

The project Joint Effort for Elimination of TB (JEET), under ‘The Global Fund to Fight AIDS, Tuberculosis and Malaria’ grant cycle 2021–2024, implemented a multi-centric TBI initiative and provision of TPT to HHCs of PTB patients diagnosed in both public and private sector. Thus, to add to the evidence towards TBI and TPT in the country, the current study aimed to document the JEET project experiences in implementing the TPT care cascade. We believe these findings will contribute to policy and practice evolutions during scaling up of TPT in India and in other countries worldwide.

## METHODS

### Study design

This is a descriptive study using individual-based patient data reported to NTEP from the project involving HHCs of patients with PTB.

### Setting

The project was implemented by FIND and implementing partners (TB Alert India and Karnataka Health Promotion Trust) in collaboration with NTEP during April 2021 to March 2024. The project facilitated management of TBI among HHCs of patients with PTB (with clinical and/or microbiological confirmation, diagnosed in public or private sector) by doorstep screening of HHCs for TB signs/symptoms including chest X-ray (wherever available) for ruling out active TB, followed by TPT initiation among eligible HHCs and provision of adherence support. The project also provided TPT awareness and facilitated adverse event (AE) management. The project data were routinely recorded in project MIS web application. The household screening was initiated from September 2021.

The study reports from 28 districts of five states (Andhra Pradesh, Karnataka, Punjab, Telangana, and Odisha) in India with a total population of ∼66.01 million. The project was operational in 12 districts during the initial phases and expanded to 28 districts (in the same states). On-ground TBI management activities were implemented in all states except Odisha, where the project provided technical assistance to support the implementation and scale-up of TPT activities. This included conducting capacity-building sessions for public and private sector providers, sharing TPT-related project materials, and supporting forecasting of diagnostic tests and drugs.

### Two project models – ‘test and treat’ and ‘treat only’

The project implemented two models: ‘test and treat’ and ‘treat only’. Under the ‘test and treat’ model, interferon-gamma release assay (IGRA) tests were offered for TBI diagnosis in HHCs (aged above 5 years). Children under 5 years followed NTEP algorithm for TPT initiation. The IGRA-positive HHCs underwent chest X-ray to rule out active TB and TPT initiation. The ‘test and treat’ model was implemented in two districts – Bagalkot (Karnataka) and Mahbubnagar (Telangana). The rest 26 districts implemented the ‘treat only’ model where no IGRA tests were carried out for TBI diagnosis.

### Implementation plan

The step-wise algorithm based on TPT guidelines is described in Figure 1.^15^ The project was implemented by dedicated district team members, namely a management information system (MIS) assistant, a tele-caller, a latent TBI coordinator, and a field officer supervised by the district lead. The HHCs of ‘test and treat’ districts received once-weekly rifapentine–isoniazid TPT regimen for 3 months (3HP) under NTEP while HHCs in other districts received once-daily isoniazid for 6 months (6H). Few HHCs in select geographies received once-daily isoniazid–rifampicin for 3-month duration (3HR) under NTEP. The 3-year project completed its activities end of March 2024. State- and district-level dissemination meetings were organised for sharing of project experiences. The project learnings were integrated into routine programme implementation and are being continued across all geographies.

**Figure 1.**
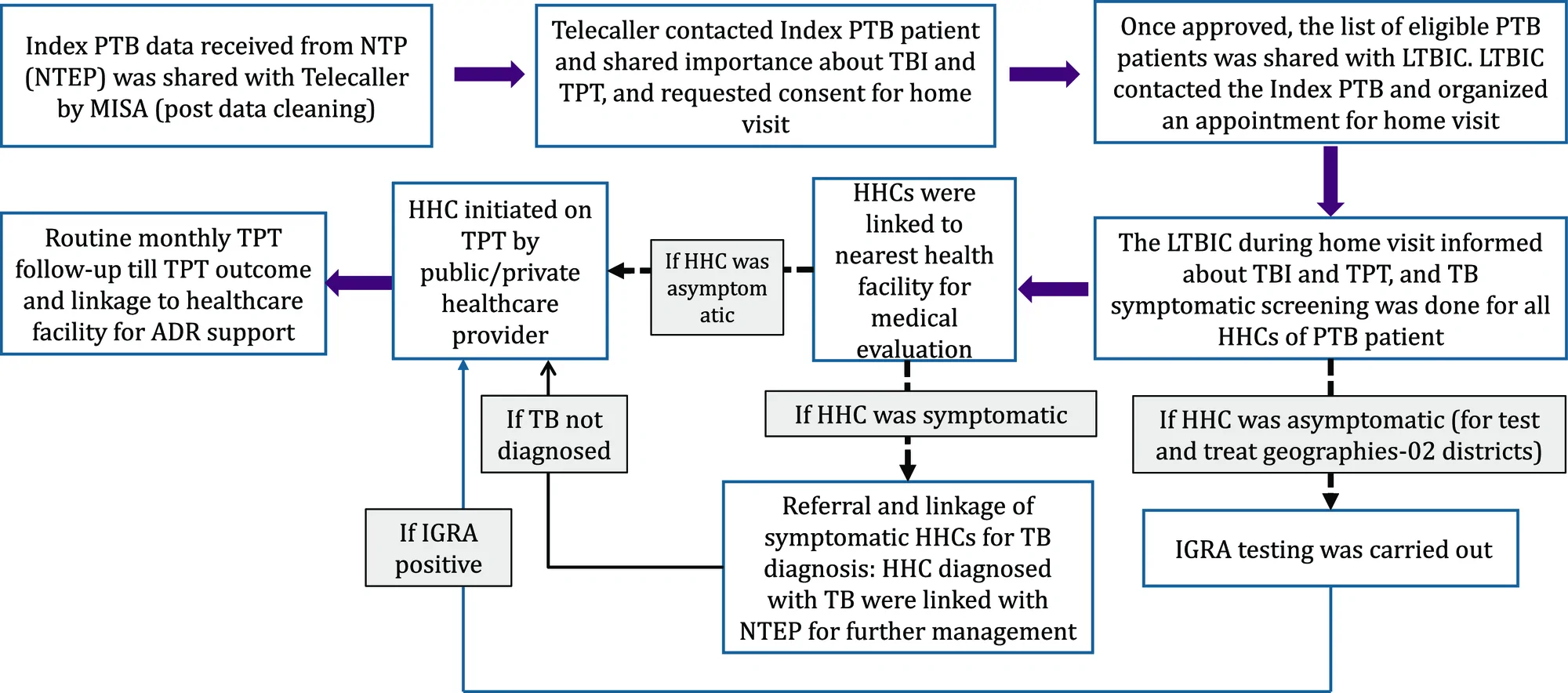
Operational cascade under JEET project, India, 2021–2024. ADR = adverse drug reaction or adverse event; PTB = pulmonary TB; NTEP = National TB Elimination Programme; TBI = TB infection; TPT = TB preventive treatment; LTBIC = latent TBI coordinator; HHC = household contact; IGRA = interferon-gamma release assay.

### Study population and participants

All HHCs of PTB patients (with either bacteriological or clinical confirmation) notified in the project geographies during September 2021 to March 2024 were included in the study.

### Data management and analysis

The source of data was JEET project MIS (electronic web-based portal). The variables from the project MIS was extracted in MS Excel format, cleaned, and imported in STATA (StataCorp LP USA) for analysis. Key analytic outputs were the number and proportion of HHCs screened, HHCs put on TPT, and HHCs who completed TPT (among those who were initiated TPT 1 year prior). The TPT outcomes were based on TPT guidelines.^15^ The categorical variables were described in proportions. Chi-square analysis (*P* value) was carried out to compare the demographic characteristics and related Index TB patient characteristics of HHCs who were screened for TB, initiated on TPT, and who did not complete TPT. Systematic recording of AE under the project was initiated in April 2022; therefore, AE analysis was limited to the period from April 2022 to March 2024. Numbers and proportions were used to summarise the AEs and TPT outcomes of HHCs.

### Ethical statement

The study was based on review of routine JEET project data, that was conducted post approval from NTEP. All data were made fully anonymous before they were accessed for the study. The extracted de-identified anonymised data were analysed for the study. The study received ethics approval from Institutional Ethics Committee, Gurushree Hi-Tech Multi Speciality Hospital, Bangalore, India (09/023/004IEC, 8 September 2023).

## RESULTS

A total of 151,890 patients with PTB were notified in the project geographies during September 2021 to March 2024. Of these, 138,812 (91%) could be contacted and 112,687 (74%) consented for home visit (Figure 2). Post consent, home visits were carried out in homes of 94,621 patients. During these home visits, 403,923 HHCs of Index PTB patients were identified, of whom 398,822 (99%) were screened for TB. Further, 310,605 (77%) HHCs consented for TPT and 253,885 (63%) were initiated on TPT.

**Figure 2.**
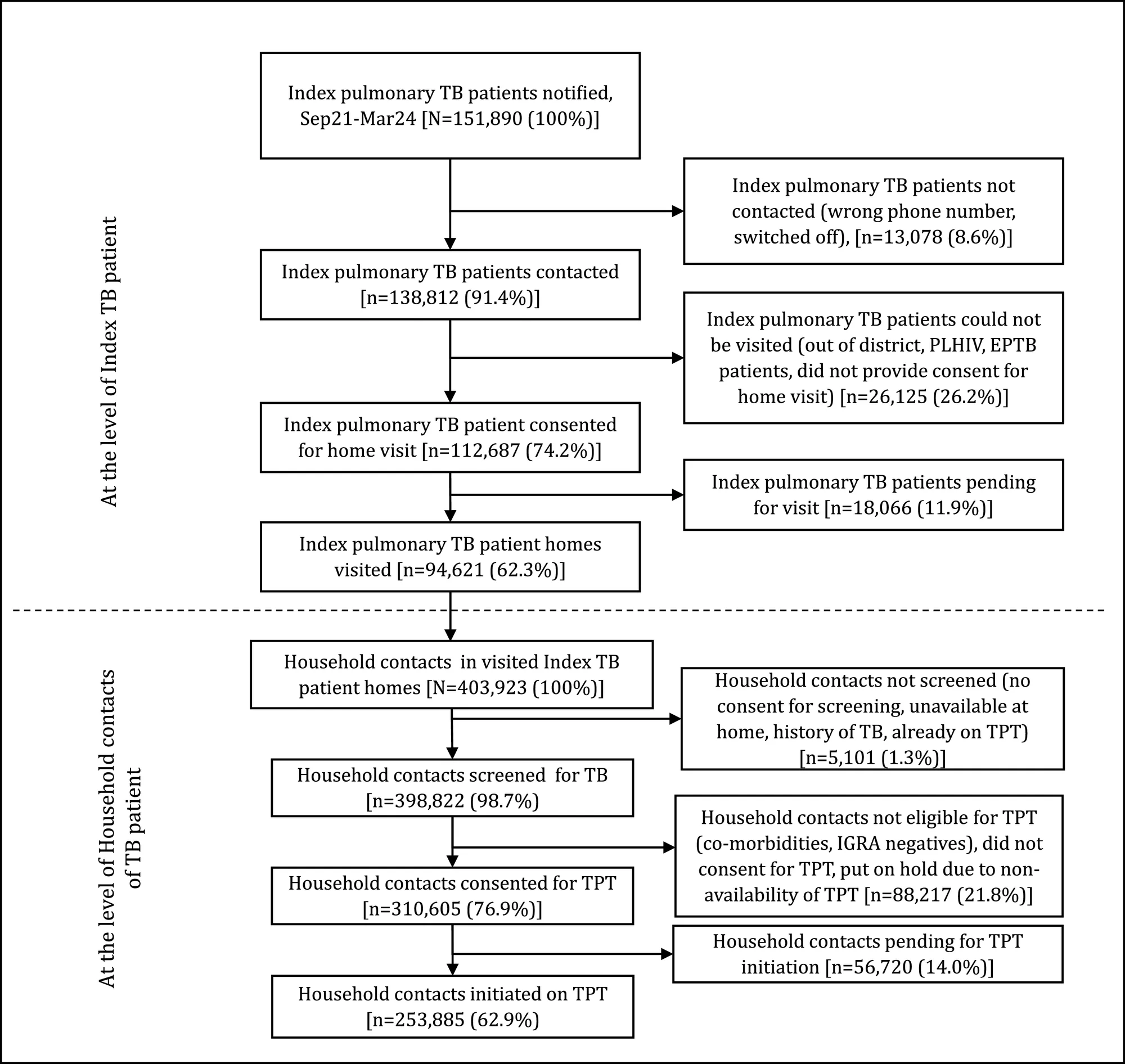
Index pulmonary TB patients home visited, household contacts screened, and initiated on TPT in project geographies in the JEET project, India, 2021–2024. PLHIV = people living with HIV; EPTB = extra-pulmonary TB; TPT = TB preventive treatment.

The demographic and clinical characteristics of the HHCs screened for TB and those initiated on TPT are presented in Table 1. Among 398,822 HHCs screened for TB, 89,650 (22%) were <15 years of age and 206,625 (52%) were women. Majority (81%, n = 321,571) of the Index TB patients received TB treatment from public sector and 66% (n = 263,539) had microbiological confirmation.

**Table 1.** Demographic and clinical characteristics of Index pulmonary TB patients and their household contacts screened for TB and those initiated on TB preventive treatment (TPT) in JEET project, India, 2021–2024.

Characteristics	Household contacts screened for TB (September 2021 to March 2024)		Household contacts initiated on TPT (September 2021 to March 2024)		*P* value
	Number	Percent**^A^**	Number	Percent^B^	
Total	398,822	(100)	253,885	(63)	
Household contact characteristics					
Age (in years)					
Less than 15	89,650	(22)	58,966	(66)	<0.001
15 and above	309,172	(78)	194,919	(63)	
Sex					
Male	192,105	(48)	121,214	(63)	0.003
Female	206,625	(52)	132,609	(64)	
Transgender	92	(<1)	62	(67)	
Geographical location					
Andhra Pradesh	119,237	(30)	75,647	(63)	<0.001
Karnataka	88,529	(22)	52,549	(59)	
Punjab	110,288	(28)	72,988	(66)	
Telangana	80,768	(20)	52,701	(65)	
Index TB patient characteristics					
TB notification sector					
Public	321,571	(81)	210,320	(65)	<0.001
Private	70,124	(18)	41,277	(59)	
Not recorded	7,127	(1)	2,288	(32)	
Type of case					
Microbiological confirmation	263,539	(66)	174,498	(66)	<0.001
Clinical confirmation	115,588	(29)	72,241	(63)	
Not recorded	19,695	(5)	4,371	(22)	
Time between Index TB patient diagnosis and household contact screening (in days, n = 363,487)					
Less than 30	184,372	(51)	120,710	(65)	<0.001
31–59	84,096	(23)	49,488	(59)	
60 and above	95,019	(26)	49,556	(52)	

A Column percentage.

B Row percentage with denominator of HHCs screened in respective category; *P* value derived with χ^2^ test.

A higher proportion of TPT initiation was reported in age group less than 15 years (66% vs. 63%, *P* < 0.001) compared to those aged 15 years and above (Table 1). The proportions of HHCs of Index TB patients receiving TB treatment from public sector (65% vs. 59%, *P* < 0.001) were also higher than those receiving TB treatment in private sector. A larger proportion (65%, *P* < 0.001) of screened HHCs were initiated on TPT whose turnaround time between date of Index TB patient diagnosis and date of HHC screening was less than 30 days compared to those with turnaround time more than 30 days.

Among 217,421 HHCs who initiated TPT during April 2022 to March 2024, 6,497 (3%) reported with AE episodes of any grade (Table 2). A higher proportion of HHCs who received 3HP developed AE episodes of any grade (8%) compared to those who received 6H or 3HR TPT regimens (3% in each group) (*P* < 0.001). Of the 6,497 HHCs that reported AE episodes, 5,778 (89%) had one episode. Majority (56% of 6,497) developed AE episodes of any grade within 15 days of TPT initiation. Gastrointestinal complaints were most common type of AE reported (63% of 7,459 AE episodes). Among 7,459 AE episodes, HHCs with 4,759 (64%) AE episodes did not require medical attention. For those who needed medical attention (36%), none had a severe AE or subjected to grave consequences.

**Table 2.** Adverse events (AEs) of any grade during TB preventive treatment (TPT) among household contacts (HHCs) who were initiated on TPT in JEET project, India, 2022–2024.

Characteristics	HHCs who received TPT (April 2022 to March 2024)		HHCs with AE episodes		*P* value
	Number	Percent**^A^**	Number	Percent**^B^**	
Total HHCs	217,421	(100)	6,497	(3)	
TPT regimen					
6H	208,880	(96)	5,837	(3)	<0.001
3HP	7,805	(4)	640	(8)	
3HR	736	(<1)	20	(3)	
HHCs with AE episodes of any grade (n = 6,497)					
One episode			5,778	(89)	
Two or more			719	(11)	
Time to first AE episode of any grade (in days, n = 6,497)					
Less than 15			3,666	(56)	
15–30			994	(15)	
31–60			898	(14)	
More than 60			939	(15)	
Types of AE**^C^** (number of episodes of any grade = 7,459)					
Gastrointestinal complaints			4,684	(63)	
Persistent fatigue			1,623	(22)	
Dark coloured urine			1,495	(20)	
Itching or rash			1,127	(15)	
Jaundice			48	(1)	
Others			410	(5)	
AE episodes of any grade requiring medical consultation**^C^** (n = 7,459)					
HHCs needed medical attention			2,690	(36)	
HHCs did not need medical attention			4,769	(64)	

A Column percentage.

B Row percentage.

C All AE episodes experienced are reported; thus, the number of AE episodes (n = 7,459) is higher than number of household contacts who experienced AEs during TPT (n = 6,497); *P* value is derived from χ^2^ test; 6H – once-daily isoniazid for 6 months as TPT; 3HP – once-weekly rifapentine–isoniazid TPT regimen for 3-month duration; 3HR – once-daily isoniazid–rifampicin for 3-month duration.

Of the 148,715 HHCs who were initiated on TPT during September 2021 to March 2023, 130,304 (88%) completed TPT. Among the rest, 12,579 (8%) were lost to follow-up (i.e., TPT interruption for ≥8 consecutive weeks for 6H and ≥4 consecutive weeks for 3HP/3HR), and 2,532 (2%) discontinued TPT due to drug toxicity. Treatment failed in 413 (<1%) HHCs while 127 (<1%) died during the TPT course. The proportions of TPT initiation showed an increasing trend from project initiation (September 2021) to September 2022; however, post September 2022, TPT initiations decreased due to interrupted availability of TPT stocks (Figure 3). A steady increase in the proportion of TPT completion among HHCs was reported during the project duration (Figure 3).

**Figure 3.**
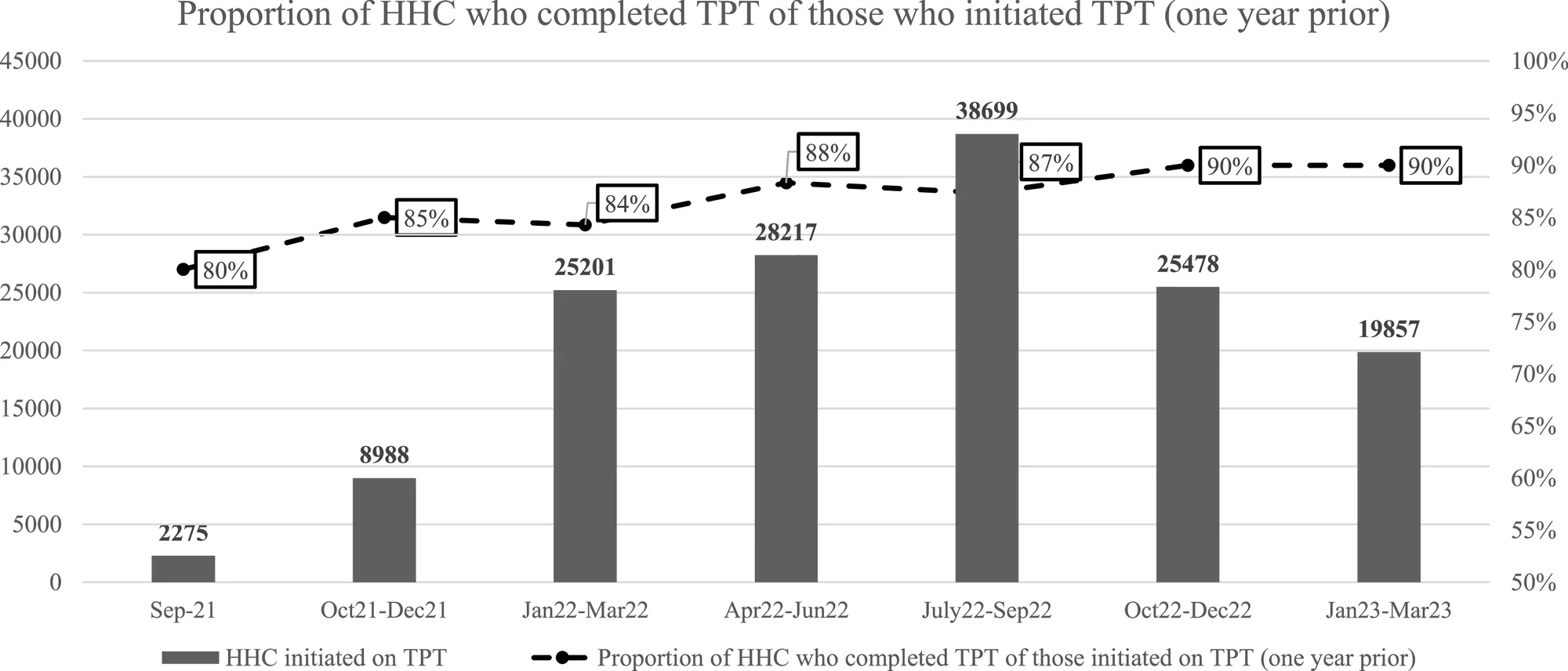
Proportion of household contacts (HHCs) who completed TPT of those who initiated TPT 1 year prior in project geographies in JEET project, India, 2021–2024. TPT = TB preventive treatment.

During JEET project implementation, multiple initiatives were carried out to strengthen the components of TBI management (detection of TBI, TPT introduction, and adherence). Awareness sessions for TBI and TPT were conducted for communities and private health care providers. Onboarding of private X-ray laboratories and video consultations for prompt medical evaluation of HHCs were carried out for early TB diagnosis and TPT initiation. Adherence mechanisms including SMS on phone, individualised zip lock pouches, and engagement of community health officers were also introduced to improve the TPT outcomes in the project.

## DISCUSSION

This study documents learnings from a large-scale implementation of TBI and TPT initiatives involving 0.4 million HHCs under routine programme settings in India. About 62% Index TB patients’ homes were visited for TB screening of HHCs. Almost all HHCs were screened for TB during home visit. The TPT was initiated among two thirds of HHCs. None of the HHCs on TPT with AE developed serious AEs, and a high proportion (88%) of TPT completion was observed under the JEET project.

More than one-third Index PTB patients’ homes could not be visited by field workers during the project duration. The reasons included incorrect patient contact details, patients moving out of district being migratory population, and perceived TB stigma leading to denial for home visits. These findings are in line with previous studies discussing challenges related to TBI and TPT implementation.^9,19,20^ However, the project team members with combined efforts of NTEP and community health workers addressed TB stigma and improved acceptability of TPT through multiple TBI awareness and counselling sessions. Therefore, addressing TB stigma and migrant population through continued involvement of community volunteers and national TB programme is essential for uptake of TBI and TPT initiatives in the vulnerable population.^21^

A significant share of HHCs screened for TB (∼37%) could not be initiated on TPT during the project tenure, which could be attributed to requirement of health care facility visit that may lead to transportation expenses and wage loss for HHCs.^22^ Other challenges could be temporary interrupted supply of TPT, hesitation for TPT (isoniazid drug resistance) among private health care providers, and no consent for TPT by HHCs due to perceived ‘good health’.^22,23^ Understanding these challenges, the project piloted tele-consultation for HHCs in remote location and periodic counselling sessions for non-consenting HHCs. One-to-one orientation and continued medical education (CME) on TBI and TPT were organised for private health care providers. Therefore, innovative context-specific strategies are needed for scaling up of TPT services in the community.

A recent systematic review on TPT regimens has reported AE during TPT^8^; however, none of the studies from India involving HHCs on TPT have reported AE in detail to our knowledge.^17–19^ In this study, 3 in 100 developed AE of any grade. Though one third of HHCs with AE needed medical attention, none reported hospitalisation. More than half HHCs reported AE within 15 days of TPT initiation, highlighting the need of closer follow-up during initial period post TPT initiation.

High treatment completion (88%) was achieved among the HHCs who initiated TPT comparable with other studies in India (77% in a study from Karnataka state, 91% in Maharashtra state).^17,19^ This study showed steady increasing trend in TPT completion rates, that could be attributed to active follow-up of HHCs during TPT duration. Missed dose monitoring was carried out by pill counting during in-person visits, AE management, and continued adherence monitoring during phone call interactions. Shorter regimen in this study had higher TPT completion rates, suggesting prioritisation of shorter TPT regimens in national programmes.^24^

Among HHCs whose Index TB patient received TB treatment from private sector, the proportions of screened HHCs initiated on TPT and those completing TPT were lower than those with Index TB patients from public sector. To strengthen the private sector involvement in TBI initiatives, CME/workshops on TBI/TPT were organised, under guidance of NTEP. A designated team member (field officer) improved liaising with private providers and early TB screening of HHCs. These initiatives improved the private sector engagement in the project, thereby highlighting the need of regular sensitisation workshops and liaising efforts for health care providers for early TBI detection and subsequently TPT initiation.^25–27^

This study documents the community-level implementation of the 3-year project, and strategies carried out to mitigate implementation challenges encountered during the project, that could help TB programmes in other low- and middle-income countries. The study had few limitations. A small proportion of Index TB patients could not be contacted. The details on comorbidities for HHCs were unavailable. Systematic recording of AE was initiated post 6-months of project implementation; therefore, AE during early phases of project could not be analysed. Long-term follow-up of HHCs post TPT completion was not carried out under routine project activities; however, a post-TPT follow-up of a subset of HHCs was reviewed under an operational research study.

## CONCLUSION

The JEET project demonstrated a community-based model for nation-wide implementation of TBI and TPT initiatives, under guidance of NTEP. These efforts, post project closure, have been sustained using domestic funding in India. Similar innovative community-based initiatives are essential in low socio-economic countries for meeting the global End TB strategy targets.
